# Club cell protein (CC16) in serum as an effect marker for small airway epithelial damage caused by diesel exhaust and blasting fumes in potash mining

**DOI:** 10.1007/s00420-023-02035-x

**Published:** 2023-12-18

**Authors:** Savo Neumann, Swaantje Casjens, Frank Hoffmeyer, Katrin Rühle, Lisa Gamrad-Streubel, Lisa-Marie Haase, Katharina K. Rudolph, Jörg Giesen, Volker Neumann, Dirk Taeger, Dirk Pallapies, Thomas Birk, Thomas Brüning, Jürgen Bünger

**Affiliations:** 1 Institute for Prevention and Occupational Medicine of the German Social Accident Insurance, Institute of the Ruhr University Bochum (IPA), Bürkle-de-la-Camp-Platz 1, 44789 Bochum, Germany; 2 Environment and Health, Ramboll Deutschland GmbH, City Tower-Limbecker Platz 1, 45127 Essen, Germany; 3 Institute for the Research on Hazardous Substances (IGF), 44789 Bochum, Germany

**Keywords:** Nitrogen oxides, Diesel particulate matter, Occupational exposure limit, Cigarette smoking, SCGB1A1, Circadian rhythm

## Abstract

**Objective:**

The effect marker club cell protein (CC16) is secreted by the epithelium of the small respiratory tract into its lumen and passes into the blood. Increased amounts of CC16 in serum are observed during acute epithelial lung injury due to air pollutants. CC16 in serum was determined as part of this cross-sectional study in underground potash miners on acute and chronic health effects from exposures to diesel exhaust and blasting fumes.

**Methods:**

Nitrogen oxides, carbon monoxide, and diesel particulate matter were measured in 672 workers at a German potash mining site on a person-by-person basis over an early shift or midday shift, together with CC16 serum concentrations before and after the respective shift. CC16 concentrations and CC16 shift-differences were evaluated with respect to personal exposure measurements and other quantitative variables by Spearman rank correlation coefficients. CC16 shift-differences were modeled using multiple linear regression. Above-ground workers as reference group were compared to the exposed underground workers.

**Results:**

Serum concentrations of CC16 were influenced by personal characteristics such as age, smoking status, and renal function. Moreover, they showed a circadian rhythm. While no statistically significant effects of work-related exposure on CC16 concentrations were seen in never smokers, such effects were evident in current smokers.

**Conclusion:**

The small airways of current smokers appeared to be vulnerable to the combination of measured work-related exposures and individual exposure to smoking. Therefore, as health protection of smokers exposed to diesel exhaust and blasting fumes, smoking cessation is strongly recommended.

## Introduction

In addition to salt dust, underground potash miners are exposed to diesel exhaust (DE) and blasting fumes (BF). In two German potash mines, a slight decrease in lung function was described in long-term underground potash miners—especially smokers—but no dose–response relation was observed (Lotz et al. 2008). In the current study, exposure and possible health effects were determined in the same mines to further elucidate the hazards of DE and BF exposure in underground potash mining. As part of the study, the effect marker club cell protein (CC16) was determined in blood serum pre- and post-shift, to evaluate acute health effects on the small airways from exposures to DE and BF during a work shift.

The use of CC16 as a serological effect marker is an approach for the early detection of adverse effects on the respiratory tract within the framework of occupational health prevention. CC16's suitability as a biomarker of acute or chronic pulmonary effects was critically reviewed before (Lakind et al. 2007).

CC16, a protein of 15.8 kDa molecular mass with a homodimer structure, is produced and secreted by club cells, epithelial, cilia-less cells of the small airways up to the terminal bronchioles. It prevents excessive inflammation in the small airways with destruction and remodeling of lung tissue, which was demonstrated in animal models of CC16-deficient mice (Laucho-Contreras et al. 2018). After secretion from club cells into the bronchioles, CC16 diffuses across the epithelial barrier into the blood, due to its small molecular size and protein nature. There was evidence that acute injury makes the epithelium more permeable (Blomberg et al. 2003; Michel et al. 2005), resulting in increased leakage of CC16 into the blood, before it is excreted by the kidneys. As a low-molecular-weight protein, CC16 is glomerularly filtered, tubularly reabsorbed, catabolized, and excreted within a few hours (Broeckaert et al. 2000; Doyle et al. 1998).

An increase in CC16 serum concentrations was observed after acute high respiratory exposures to pollutants from fire gases (Krakowiak et al. 2013), and a reduction in serum was described in subjects with lung damage after chronic exposure to air pollutants or in long-term smokers (Broeckaert et al. 2000). Reduced CC16 concentrations in serum were also described in various lung diseases (Li et al. 2019) such as asthma (Zhu et al. 2019; Stenberg et al. 2018) and COPD (Guerra et al. 2015) compared to healthy nonsmokers. In allergic events, CC16 was attributed a modulatory role (Hung et al. 2004; Tafuro et al. 2018). Besides an anti-inflammatory effect, an antitumor effect was also discussed (Lakind et al. 2007). A reduction of CC16 occurs as a consequence of altered gene expression by smoking (Zhu et al. 2015; Buro-Auriemma et al. 2013) and also after methylation of DNA by exposure to DE (Wang et al. 2019b). Club cells themselves still possess stem cell potential for dedifferentiation and redifferentiation (Wang et al. 2019a; Zuo et al. 2018).

A time dependence of serum CC16 concentrations during the course of the day (Helleday et al. 2006) and, also, the role of epithelial circadian control of inflammation localized in club cells (Gibbs et al. 2014; Ince et al. 2019) were described.

The objective of this part of the current health study was to determine whether acute damaging effects of exposures on the small airways could be identified during a work shift. Therefore, CC16 and the personal exposures to nitrogen oxides and inorganic particulate matter measured as elemental carbon (EC-DPM) from DE in underground workers were compared with the above-ground workers as a reference group. Because of the large cohort working in different shifts and the measurement of further parameters such as serum creatinine, carboxyhemoglobin, and cotinine, it was possible to further evaluate these factors with respect to the suitability of CC16 as effect marker under field conditions.

## Materials and methods

The ethics application was approved by the Ruhr University Bochum under registration no. 17-6024. All participants of the study gave written informed consent according to the Declaration of Helsinki in its current edition.

### Study population

The study in German potash mining was recently described (Gamrad-Streubel et al. 2022). A requirement for inclusion was employment in potash mining for at least one year in the facility above ground or in the mine underground. CC16 concentrations from one investigated mining site were further analyzed. In this analysis, a total of 689 employees were considered, who were examined in the years 2017–2018. Due to the small proportion of 6 female employees, their data were not included in the analysis. Of the remaining 683 male employees, 672 were evaluated as the study population, because pre- and post-shift CC16 values were available for them.

### Working conditions

All employees above ground in the facility and underground in maintenance and mining worked in shifts. Operations in the facility run 24 h a day, 7 days a week in a fully continuous shift system. Maintenance and mining employees worked from Sunday night shift to Saturday midday shift, with weekly shift changes from early to midday to night shift. Only early and midday shift workers were studied. The early shift (ES) extended from 5 a.m. to 1 p.m. and the midday shift (MS) from 1 p.m. to 9 p.m. Workers underground were exposed to dry heat with temperatures of up to 41 °C and relative air humidity of 22% in mining areas and up to 28 °C and 43% relative humidity in maintenance areas. These conditions were measured in 2017 at typical working places, while testing the equipment for person-by-person measurement under field conditions.

### Smoking status

Anamnestic data on smoking status and consumption of tobacco products were collected by written questionnaire. A distinction was made between never smokers, former smokers, occasional smokers (up to a maximum of 5 cigarettes per week), and current smokers. The data on consumption of tobacco products included cigarettes, cigars, cigarillos, and tobacco pipes and were converted into cigarette equivalents (Latza 2005). The nicotine metabolite cotinine was determined from urine pre- and post-shift as exposure marker for smoking, as well as carboxyhemoglobin (COHb) from blood (expressed as percent of hemoglobin) as an exposure marker for CO.

### Atopy status

Determination of Cap IgE sx1 was used to determine whether an atopic disposition was present in employees (van Kampen et al. 2020). This was assumed when Cap IgE sx1 ≥ 0.35 kU/l was measured pre-shift. Cap IgE sx1 was determined from serum using the method of Fluorescent Enzyme Immunoassay (FEIA) with the analyzer ImmunoCap Phadia™ 1000 (ThermoFisher Scientific).

### Exposure measurements and group comparison

The personal exposure measurements were carried out on-site by experts from the Institute for the Research on Hazardous Substances (IGF) of the Employer's Liability Insurance Association for Raw Materials and the Chemical Industry (BG RCI). During a work shift, EC-DPM was collected person-related by means of PDS-A dust collectors driven by a pump of the type SG 5100ex with 2 l/min-throughput (GSA Messgerätebau, Germany), then determined gravimetrically and coulometrically in the laboratory of the IGF and given as a shift mean value in mg/m^3^ for an 8-h work shift. At the same time, the personal exposures for NO, NO_2_, and CO were measured in ppm with direct-reading gas measuring instruments ‘Dräger X-am^®^ 5600’ (Lübeck, Germany) and given also as 8-h shift mean values, as well as 15-min peak values. The individual workplace exposures to DE and BF, respective NO, NO_2_, CO, and EC-DPM, cannot be considered independent (Backé et al. 2004; Dahmann et al. 2007; Lotz et al. 2008) as for technical reasons they are highly correlated and occur as a mixture in the mine. Therefore, the exposure situation of the workers was summarized by the group comparison between the three differently exposed groups, namely facility (reference) and the exposed groups of maintenance and mining. The German occupational exposure limits (OEL) are 2.0 ppm for NO, 0.5 ppm for NO_2_, 30 ppm for CO, and 0.05 mg/m^3^ for diesel particulate matter measured as EC-DPM, but for NO, NO_2_, and EC-DPM, they are not yet enforced in underground mining by regulatory exception until the 21st August of 2025 (BMAS Federal Ministry of Labour and Social Affairs 2023).

### Medical examinations and CC16

Study participants underwent medical examinations with laboratory analyses of blood and urine. Serum creatinine (normal ≤ 1.2 mg/dl) was determined enzymatically using the analyzer cobas® 8000 (Roche Diagnostics). Samples were drawn Monday through Friday immediately before and after their work shift in parallel to the personal exposure measurements. Subjects were interviewed and examined by medical personnel, which was employed and trained specifically for the study and located in examination containers, set up directly at the mining site. Body height and weight were measured once before the work shift. The timepoints of blood sampling, urine collection and examinations were documented. Samples were processed immediately according to the reference manual of the external medical laboratory, frozen at − 20 °C if necessary, and transported to the laboratory daily by courier service.

CC16 concentrations were determined from serum, using the Human Clara Cell Protein (CC16) ELISA kit (CUSABIO®, Wuhan, China). To date, no diagnostic reference value for CC16 in serum is established. Detection limits were reported by the medical laboratory as 1.6–400 ng/ml. In 13% measurements were below limit of detection (LOD). Determination above 400 ng/ml was possible by dilution in one subject. CC16 is also referred to as SCGB1A1 according to its assignment to the secretoglobin family, or formerly as uteroglobin, CC10 or CCSP. The CC16 shift-differences, formed by CC16 concentrations post-shift minus CC16 concentrations pre-shift, reflected the acute exposure effect on the small airways over the work shift.

### Statistical methods

Continuous variables were characterized using medians and interquartile ranges (IQR). Boxplots with median, IQR, and whiskers representing the minimum and maximum were used to show the distribution of CC16. Location differences were examined using the non-parametric Wilcoxon–Mann–Whitney test or Kruskal–Wallis test for independent samples. Wilcoxon sign rank tests were used to compare pre- and post-shift values. *χ*^2^ test was used to detect dependencies between two categorical characteristics. The distributions of personal exposure measurements were presented using cumulative distribution functions stratified by group, and the proportion of subjects below the OEL was shown. Associations between CC16 concentrations and CC16 shift-differences with personal exposure measurements and other quantitative measures were presented by Spearman rank correlation coefficients (r_S_) with 95% confidence intervals (95% CI) and *p* values. EC-DPM could not be determined in 8 subjects, and in 145 subjects, the EC-DPM measurements were below the limit of quantification (LOQ). At a sampling rate of 2 l/min and an 8-h shift, the LOQ for EC-DPM was 0.025 mg/m^3^. We treated values below LOD (CC16) and LOQ (EC-DPM) as left-censored, where the LOD or LOQ represented the maximum possible value. Values below LOD or LOQ were imputed 1000 times at random from a log-normal distribution (Lotz et al. 2013). We assessed the influence of exposure on CC16 shift-differences using linear regression models that included multiple imputed CC16 values below the LOD and an adjustment set consisting of shift, atopy, post-shift creatinine, age, and smoking status. Statistical analyses were performed using SAS, version 9.4 (SAS Institute Inc., Cary, NC, USA) and figures were generated using GraphPad Prism, version 9 (GraphPad Software, La Jolla, California, USA).

## Results

### Characterization of groups by conditions of exposure, shift, age, atopy, and smoking status

The distribution of groups between the two shifts (ES and MS) was significantly different (*p* < 0.0001). In the facility, employees were distributed by 70% (*n* = 68) to ES and 30% (*n* = 29) to MS, in maintenance by 65% (*n* = 63) to ES and 35% (*n* = 34) to MS, and in mining equally by 50% (*n* = 237) to ES and (*n* = 241) MS, respectively. Age distribution differed between work shifts (ES 38.5 years, IQR 31–48; MS 36.5 years, IQR 30–47, *p* = 0.0129). Further, the groups also differed by age. The maintenance group, with a median age of 44 years (IQR 33–52), was older than the facility group with 38 years (IQR 31–49) and the mining group with 37 years (IQR 30–47) (*p* = 0.0006).

286 employees were serologically classified as atopic and 386 as non-atopic with reference to sx1.

The distribution of the 672 workers in total and stratified by work shift and group is shown in Table 1. Above ground, 97 study participants worked in the facility, whereas 575 worked underground, of which 97 belonged to the maintenance group and 478 to the mining group.

**Table 1:** Description of the cohort (*n* = 672) studied at the potash mining site

		Total	Facility (*n* = 97)	Maintenance (*n* = 97)	Mining (*n* = 478)	Early shift (*n* = 368)	Midday shift (*n* = 304)
Age [years]	Median (IQR)	37 (30–47)	38 (31–49)	44 (33–52)	37 (30–47)	38.5 (31–48)	36.5 (30–47)
Body height [cm]	Median (IQR)	181 (177–186)	182 (177–185)	181 (176–186)	181 (177–185)	182 (177–186)	181 (176–185.5)
Weight [kg]	Median (IQR)	90 (81.9–100.1)	91.6 (80.5–104.0)	88.8 (80.5–100.0)	89.8 (82.0–100.0)	89.7 (80.6–99.7)	90 (82.7–102.0)
Duration of employment [years]	Median (IQR)	11.3 (6.6–19.4)	10.2 (6.6–21.5)	11.5 (6.9–21.3)	11.4 (6.5–19.2)	11.4 (6.9–19.8)	11.3 (6.3–18.9)
Group (*N*, %)	Facility	97 (14.4)	97 (100)			68 (18.5)	29 (9.5)
	Maintenance	97 (14.4)		97 (100)		63 (17.1)	34 (11.2)
	Mining	478 (71.1)			478 (100)	237 (64.4)	241 (79.3)
Smoking status (*N*, %)	Never	238 (35.4)	37 (38.1)	30 (30.9)	171 (35.8)	136 (37)	102 (33.6)
	Former	137 (20.4)	22 (22.7)	24 (24.7)	91 (19.0)	76 (20.7)	61 (20.1)
	Occasional	64 (9.5)	7 (7.2)	11 (11.3)	46 (9.6)	31 (8.4)	33 (10.9)
	Current	231 (34.4)	31 (32.0)	32 (33.0)	168 (35.1)	124 (33.7)	107 (35.2)
	No data	2 (0.3)				1 (0.27)	1 (0.33)
Atopy (sx1 ≥ 0.35 kU/l) (*N*, %)	Yes	286 (42.6)	36 (37.1)	38 (39.2)	212 (44.4)	159 (43.2)	127 (41.8)
	No	386 (57.4)	61 (62.9)	59 (60.8)	266 (55.6)	209 (56.8)	177 (58.2)

The study population included 238 never smokers, 137 former smokers, 64 occasional smokers, 231 current smokers, and 2 subjects of unknown smoking status. For 227 participants, anamnestic data about smoking consumption were available. Current smokers in the facility group had a statistically significant higher median daily smoking consumption of 17.5 (IQR 11–20) cigarette equivalents compared to 10 (IQR 6.5–15) in the maintenance group and to 10 (IQR 8–15) in the mining group (*p* = 0.0001). For the nicotine metabolite cotinine, pre-shift values were available for 651 workers, of which 381 were below the LOD of 2 µg/l with a median of < 2 µg/l (IQR < 2–698). Post-shift, values were available for 659 workers, of whom 390 were below the LOD with a median of < 2 µg/l (IQR < 2–580).

**Fig. 1:**
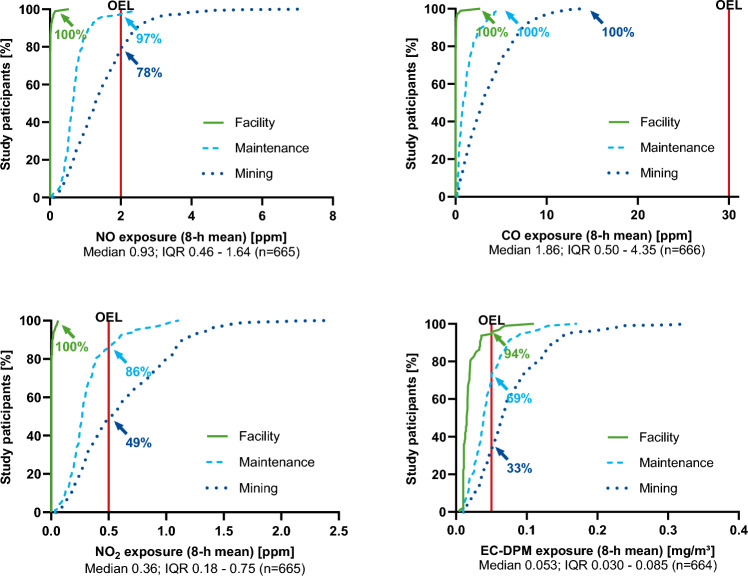
Percentage of study participants by group and exposure to NO, NO_2_, CO, and EC-DPM by 8-h mean. Respective occupational exposure limits (OEL) are shown. Medians and interquartile ranges (IQR) represent the entire study population

For comparison of the three groups, the facility served as reference group due to the lowest personal exposures to NO, NO_2_, CO, and EC-DPM, followed by maintenance workers with medium exposures and miners with highest exposures (Fig. 1). In the facility, 8-h mean concentrations for NO, NO_2_, and CO lay below OEL and close to zero for 100% of the workers and for EC-DPM 94% of the values below OEL. In miners, exposures to EC-DPM were below OEL for only 33% of the workers. The percentage of study participants exposed below OEL can also be seen in Fig. 1.

### CC16 showed a circadian rhythm

**Fig. 2:**
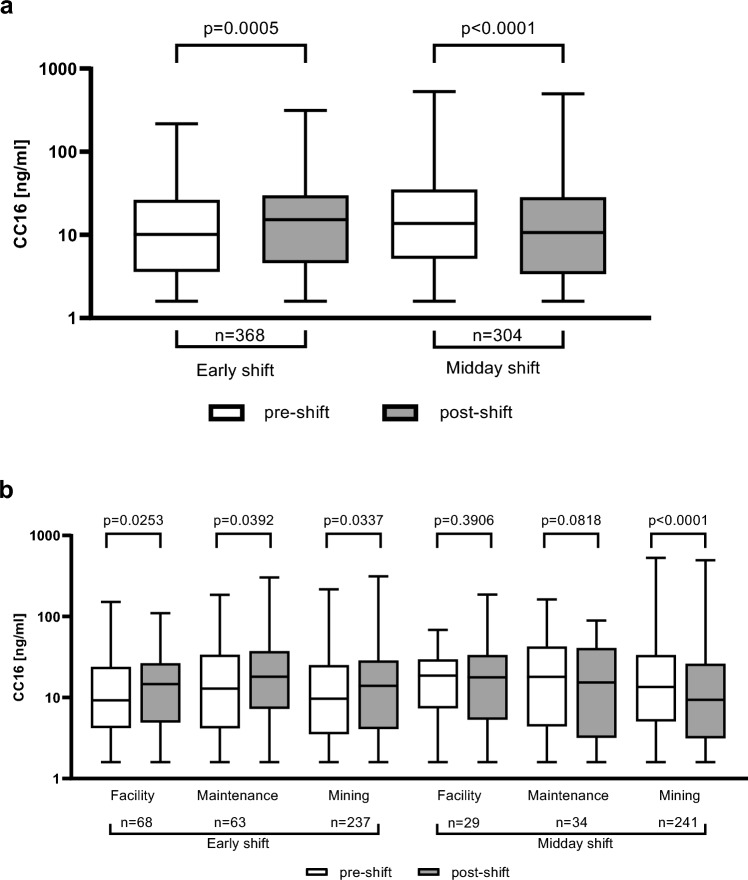
**a** Boxplots of CC16 concentrations pre- and post-early shift (ES) and midday shift (MS). **b** Boxplots of CC16 concentrations for exposition groups pre- and post-early shift (ES) and midday shift (MS)

In both work shifts together (ES and MS), the median values for serum CC16 concentrations pre-shift were 12.15 ng/ml (IQR 4.25–29.15) and post-shift 12.65 ng/ml (IQR 4.1–29.2, p = 0.7058). Analyzing shifts separately, medians were lower in the morning pre-ES with 10.15 ng/ml (IQR 3.65–26.00) than in the noon post-ES with 15.30 ng/ml (IQR 4.60–29.70, p = 0.0005). Across MS, medians trended in opposite direction and were higher pre-MS with 13.80 ng/ml (IQR 5.20–34.80) than in the evening post-MS with 10.70 ng/ml (IQR 3.40–28.10, *p* < 0.0001). CC16 concentrations in serum showed a circadian rhythm with a midday peak (post-ES and pre-MS) as depicted in Fig. 2a and for exposition groups shown in Fig. 2b.

### CC16 concentrations but not CC16 shift-differences increased with age

With increasing age, CC16 concentrations were positive correlated both pre- and post-shift (pre-shift r_S_ = 0.14, 95% CI 0.07–0.21, *p *= 0.0003; post-shift r_S_ = 0.13, 95% CI 0.06–0.21, *p* = 0.0006). There was no statistically significant correlation with age for the CC16 shift-differences (r_S_ = 0.00, 95% CI − 0.08 to 0.07, *p* = 0.9005).

### Carboxyhemoglobin (COHb) decreased for current smokers underground

Current smokers in the facility group showed no significant change in median COHb across the work shift (pre 5.70%, IQR 4.20–6.90, post 5.90%, IQR 4.20–7.60, *p* = 0.1308) when comparing their median COHb pre-shift to post-shift. In contrast, among current smokers in underground workers, COHb decreased across the shift (maintenance: pre 5.55%, IQR 4.15–6.75, post 4.30%, IQR 3.50–4.60, *p* < 0.0001, mining: pre 6.35%, IQR 5.10–7.70, post 4.60%, IQR 4.00–5.10, *p* < 0.0001). Never smokers in the facility group showed a slight statistically significant decrease in median COHb across the shift (pre 3.40%, IQR 3.20–3.70, post 3.30%, IQR 3.00–3.50, *p* = 0.0052) when comparing their median COHb pre-shift to post-shift. Among never smokers in underground workers, a slight increase in median COHb during the shift was observed, but it was only significant in miners (maintenance: pre 3.40%, IQR 3.10–3.70, post 3.50%, IQR 3.30–3.80, *p* = 0.1378, mining: pre 3.50%, IQR 3.30–3.80, post 3.60%, IQR 3.40–4.10, *p* = 0.0015).

### CC16 shift-differences for workers underground correlated negatively with post-shift serum creatinine

Median serum creatinine for all workers was 1.0 mg/dl (IQR 0.9–1.1) pre-shift and 1.0 mg/dl (IQR 1.0–1.1) post-shift. CC16 shift-differences both for never smokers and current smokers correlated negatively with the post-shift serum creatinine levels (Table 2, 3), which were also a statistically significant negative estimator in the model (Table 4). Differentiated by group, no statistically significant correlations of post-shift serum creatinine with CC16 shift-differences (r_S_ = − 0.12; 95% CI − 0.32 to 0.08; *p* = 0.2313) were observed in the facility workers. In contrast, for those working underground, CC16 shift-differences had statistically significant negative correlation with post-shift serum creatinine (maintenance r_S_ = − 0.26; 95% CI − 0.44 to − 0.07; *p* = 0.0096 and mining r_S_ = − 0.10; 95% CI − 0.19 to − 0.01; *p* = 0.0262).

**Table 2:** Spearman correlations (r_S_) and 95% confidence intervals (CI) of CC16 concentrations pre- and post-shift in never smokers

	Pre-shift CC16 [ng/mL]				Post-shift CC16 [ng/mL]				CC16 [ng/mL] (post–pre)			
	r_S_	95% CI		*p* value	r_S_	95% CI		*p* value	r_S_	95% CI		*p* value
Post-shift CC16 [ng/mL]	**0.76**	**0.71**	**0.81**	** < 0.0001**								
Age [years]	0.03	− 0.10	0.16	0.6546	0.07	− 0.06	0.20	0.2871	0.02	− 0.10	0.15	0.7112
Weight [kg]	0.06	− 0.07	0.18	0.3775	0.07	− 0.06	0.19	0.3071	0.09	− 0.04	0.22	0.1612
Body height [cm]	0.03	− 0.09	0.16	0.5973	0.01	− 0.12	0.13	0.9276	0.02	− 0.10	0.15	0.7231
Duration of employment [years]	0.04	− 0.09	0.16	0.5752	0.04	− 0.09	0.16	0.5690	− 0.03	− 0.16	0.10	0.6571
Pre-shift creatinine [mg/dl]	0.01	− 0.12	0.14	0.8805					− 0.09	− 0.21	0.04	0.1897
Post-shift creatinine [mg/dl]					− 0.02	− 0.15	0.11	0.7435	**− 0.15**	**− 0.27**	**− 0.03**	**0.0183**
NO exposure (8-h mean) [ppm]	0.02	− 0.11	0.14	0.8140	0.05	− 0.07	0.18	0.4047	0.03	− 0.09	0.16	0.5970
NO exposure (15-min peak) [ppm]	0.01	− 0.12	0.14	0.8533	0.08	− 0.04	0.21	0.1962	0.07	− 0.06	0.19	0.3153
NO_2_ exposure (8-h mean) [ppm]	− 0.05	− 0.18	0.08	0.4620	− 0.03	− 0.15	0.10	0.7034	0.03	− 0.10	0.16	0.6124
NO_2_ exposure (15-min peak) [ppm]	− 0.09	− 0.21	0.04	0.1752	− 0.07	− 0.20	0.06	0.2679	0.02	− 0.11	0.14	0.7980
CO exposure (8-h mean) [ppm]	− 0.01	− 0.14	0.11	0.8299	0.03	− 0.10	0.16	0.6569	0.04	− 0.08	0.17	0.5024
CO exposure (15-min peak) [ppm]	− 0.02	− 0.15	0.10	0.7109	0.05	− 0.08	0.18	0.4425	0.09	− 0.04	0.22	0.1594
EC-DPM exposure (8-h mean) [mg/m^3^]	0.00	− 0.13	0.13	0.9606	0.05	− 0.08	0.18	0.4380	0.05	− 0.08	0.18	0.4509
Pre-shift carboxyhemoglobin [%]	0.04	− 0.09	0.17	0.5498					− 0.09	− 0.22	0.04	0.1639
Post-shift carboxyhemoglobin [%]					− 0.01	− 0.13	0.12	0.9105	− 0.13	− 0.25	0.00	0.0538

Values shown in bold indicate statistical significance at the *p* < 0.05 level

**Table 3:** Spearman correlations (r_S_) and 95% confidence intervals (CI) of CC16 concentrations pre- and post-shift in current smokers

	Pre-shift CC16 [ng/mL]				Post-shift CC16 [ng/mL]				CC16 [ng/mL] (post–pre)			
	r_S_	95% CI		*p* value	r_S_	95% CI		*p* value	r_S_	95% CI		*p* value
Post-shift CC16 [ng/mL]	**0.70**	**0.63**	**0.76**	** < 0.0001**								
Age [years]	**0.21**	**0.08**	**0.33**	**0.0012**	**0.17**	**0.04**	**0.29**	**0.0118**	− 0.06	− 0.19	0.07	0.3634
Weight [kg]	0.11	− 0.02	0.23	0.1051	0.05	− 0.08	0.18	0.4253	**− 0.14**	**− 0.27**	**− 0.01**	**0.0323**
Body height [cm]	− 0.02	− 0.15	0.11	0.7798	0.02	− 0.11	0.15	0.7914	0.02	− 0.11	0.15	0.8133
Duration of employment [years]	0.09	− 0.04	0.22	0.1831	**0.15**	**0.02**	**0.28**	**0.0201**	0.10	− 0.03	0.22	0.1481
Duration of smoking [years]	**0.20**	**0.07**	**0.33**	**0.0021**	**0.21**	**0.08**	**0.33**	**0.0018**	− 0.01	− 0.14	0.12	0.9093
Daily cigarette equivalents	0.03	− 0.10	0.16	0.6948	**0.14**	**0.01**	**0.26**	**0.0416**	0.13	0.00	0.26	0.0526
Pack years (cigarette equivalents)	0.12	− 0.01	0.25	0.0812	**0.20**	**0.07**	**0.32**	**0.0029**	0.09	− 0.04	0.22	0.1739
Pre-shift creatinine [mg/dl]	− 0.04	− 0.17	0.09	0.5029					− 0.04	− 0.17	0.09	0.5269
Post-shift creatinine [mg/dl]					− 0.04	− 0.17	0.09	0.5380	**− 0.16**	**− 0.29**	**− 0.03**	**0.0133**
NO exposure (8-h mean) [ppm]	− 0.10	− 0.22	0.03	0.1435	**− 0.20**	**− 0.32**	**− 0.07**	**0.0023**	− 0.10	− 0.22	0.04	0.1533
NO exposure (15-min peak) [ppm]	− 0.03	− 0.16	0.10	0.6759	**− 0.14**	**− 0.27**	**− 0.01**	**0.0314**	**− 0.13**	**− 0.26**	**0.00**	**0.0470**
NO_2_ exposure (8-h mean) [ppm]	− 0.09	− 0.22	0.04	0.1871	**− 0.18**	**− 0.30**	**− 0.05**	**0.0068**	− 0.09	− 0.21	0.04	0.1958
NO_2_ exposure (15-min peak) [ppm]	− 0.12	− 0.25	0.01	0.0623	**− 0.20**	**− 0.32**	**− 0.07**	**0.0027**	− 0.04	− 0.17	0.09	0.5335
CO exposure (8-h mean) [ppm]	− 0.06	− 0.19	0.07	0.3955	**− 0.14**	**− 0.27**	**− 0.01**	**0.0292**	− 0.09	− 0.22	0.04	0.1841
CO exposure (15-min peak) [ppm]	− 0.07	− 0.20	0.06	0.2891	− 0.12	− 0.25	0.01	0.0679	− 0.02	− 0.15	0.11	0.7435
EC-DPM exposure (8-h mean) [mg/m^3^]	− 0.08	− 0.20	0.06	0.2569	**− 0.18**	**− 0.30**	**− 0.05**	**0.0067**	− 0.12	− 0.24	0.01	0.0802
Pre-shift carboxyhemoglobin [%]	**0.16**	**0.03**	**0.29**	**0.0136**					− 0.06	− 0.19	0.07	0.3798
Post-shift carboxyhemoglobin [%]					0.06	− 0.07	0.19	0.3438	0.05	− 0.08	0.18	0.4418

Values shown in bold indicate statistical significance at the *p* < 0.05 level

**Table 4:** Multiple linear regression model for CC16 shift-differences (post-shift–pre-shift)

	*N*	*β*	95% CI		*p* value
Intercept	669	37.67	19.34	56.00	
Shift					
Early shift (reference)	367	0			
Midday shift	302	**− 5.34**	**− 9.38**	**− 1.29**	**0.0097**
Group					
Facility (reference)	97	0			
Maintenance	97	− 1.80	− 9.05	5.46	0.6277
Mining	475	− 2.23	− 7.94	3.47	0.4426
Atopy (sx1 ≥ 0.35 kU/l)					
No (reference)	384	0			
Yes	285	− 3.26	− 7.24	0.73	0.1094
Creatinine [mg/dl] (post)	669	**− 25.74**	**− 40.44**	**− 11.04**	**0.0006**
Smoking status					
Never (reference)	238	0			
Former	137	− 1.21	− 6.70	4.29	0.6669
Occasional	63	− 2.55	− 9.72	4.62	0.4862
Current	231	− 1.10	− 5.81	3.61	0.6469
Age [by 10 years]	669	− 1.06	− 3.02	0.90	0.2907

The multiple model includes data from 669 subjects for whom values were available for all influencing parameters considered. In total, data from three subjects are not included due to missing smoking status (*n* = 2) or missing creatinine value (*n* = 1)

*CI* confidence interval

### Exposures showed no effects on CC16 in never smokers but in current smokers

No statistically significant correlations of exposures and CC16 concentrations were shown for never smokers (Table 2). In contrast, for current smokers (Table 3), statistically significant negative post-shift correlations were observed with work-related exposures to NO, NO_2_, CO, and EC-DPM. Also, statistically significant positive correlations with CC16 concentrations (Table 3) were shown for age, duration of employment (only post-shift), duration of smoking, smoking consumption in daily cigarette equivalents (only post-shift), and pack-years (only post-shift). Concerning the CC16 shift-differences, for current smokers statistically significant negative correlation was shown with NO (15-min peak) and with EC-DPM (8-h mean) at least a negative trend.

### Creatinine and work shift significantly affected CC16 shift-differences

Multiple linear regression showed no association between exposure group and CC16 shift-differences after adjusting for smoking status and other factors (Table 4). However, statistically significant influences of post-shift serum creatinine and shift (ES/MS) on CC16 shift-differences were observed.

## Discussion

CC16 is a product of club cells in the epithelium of the small airways and plays an important role in preventing excessive inflammatory responses and associated cellular and epithelial damage in the lung. Inflammation can be caused by exposures to DE and BF containing nitrogen oxides (NO_X_) and/or particles (EC-DPM). Our results show changes in the effect marker CC16 in serum among DE- and BF-exposed workers in underground potash mining depending substantially on their smoking habits. The circadian variations in serum concentrations of CC16 and their dependence on renal excretion, as well as high temperatures underground, complicated the assessment of exposure effects.

### Exposures and smoking

In today’s German underground potash mining, the exposures to NO_X_ and EC-DPM are lower than in earlier studies, because more effective technical measures for reduction of work-related exposures have been successfully implemented (Dahmann et al. 2007; Gamrad-Streubel et al. 2022). Therefore, a comparison with the results of these past studies is difficult. Although the reduced OELs for NO_X_ and EC-DPM have not yet been fully undercut in mining and maintenance, the measured exposures underground showed no adverse effects on the small airways respective on the effect marker CC16 in serum. Compared to smoking, work-related CO was only a marginal source of individual exposure to CO, which was clearly shown by the low personal measured values of CO and especially by the effect marker COHb over the work shift.

Statistically significant correlations between person-specific measured occupational exposures (NO, NO_2_, CO, and EC-DPM) and post-shift CC16 concentrations were evident for current smokers. A correlation was also found between the short-term NO (15-min peak) and the CC16 shift-differences. In general, exposure to DE drives airway inflammation as shown in a controlled human exposure study (Xu et al. 2013). Our results indicate a possibly increased vulnerability to work-related exposures to NO, NO_2_ and EC-DPM for current smokers. The correlations of CC16 concentrations pre- and post-shift in current smokers with the parameters duration of employment, duration of smoking, daily cigarette equivalents, and pack-years, reflect possible chronic influences. These parameters depend on age, which is also true for serum creatinine, as renal function is known to decline with age (Broeckaert et al. 2000; Doyle et al. 1998). The facility reference group, i.e., above-ground current smokers had the highest smoking consumption, probably due to the fact that they could continue smoking during breaks, while smoking underground is prohibited for reasons of fire and explosion protection. This eliminated an acute damaging effect from smoking on the epithelium of the small airways, at least for the duration of the work shift underground. In addition, the comparison of the exposure marker COHb before and after the work shift showed, that smoking was the relevant source of exposure to CO. The number of values below the LOD for cotinine was consistent with the history of smoking status. To our knowledge, these parameters have not previously been used to objectify smoking status in comparable studies of potash mining (Backé et al. 2004; Lotz et al. 2008).

### Dry air, heat, and post-shift serum creatinine

Employees underground were exposed to dry heat, stronger in mining than in maintenance. Increased evaporation and sweating lead to high drinking requirements influencing urinary excretion and thereby CC16 concentrations in serum. This was evidenced by the statistically significant negative correlation of CC16 shift-differences with post-shift serum creatinine for underground maintenance and mining employees, as well as the significant negative estimator in the model. It has been described for patients with acute respiratory failure that the renal clearance of CC16 is related to serum creatinine (Doyle et al. 1998).

In two studies, athletes were exposed to physical exertion under different climatic conditions and the effects on CC16 concentrations in plasma were investigated. In one of these studies, 8 competitive athletes, loaded on a treadmill for 10 km in a climatic chamber at 6 °C ambient temperature at two different times of the day at 9 a.m. and 4 p.m., were examined (Boukelia et al. 2017). An increase of CC16 concentrations in plasma was described after exercise. In another study, endurance exercise of 13 subjects was performed in a climatic chamber at 28 °C and 70% humidity at 9 a.m. and 6 p.m. with increased CC16 concentrations after exercise (Boukelia et al. 2018). In comparison between these two studies, the increase was more pronounced at the lower temperature of 6 °C, which was attributed to the dryness of the cold air.

### Circadian rhythm

In these two studies, a different magnitude in increase of CC16 concentrations in plasma was described for both time points, but no circadian pattern was detected (Boukelia et al. 2017, 2018). However, we detected a circadian rhythm for CC16 concentrations in serum. This discovery was made possible by medical examination of shift workers in different shifts before and after ES or MS. This proved as crucial, to avoid misinterpreting circadian rhythmic increases and decreases of CC16 during the day as exposure effects. In two comparable studies in potash mining, there was little information on timing of sample collection or work shifts. One study did not provide any information on work shifts (Lotz et al. 2008) and the other provided blood sampling times of 6 a.m.–2 p.m. (Backé et al. 2004). The possible influence of the time of sample collection on CC16 concentrations in serum was specifically investigated only for small study populations so far. A total of 18 healthy never smokers, 5 males and 13 females, were examined between 7 a.m. and 10 p.m. with 6 blood samples each (Helleday et al. 2006). This procedure was repeated on two further dates weeks apart. A correction formula was proposed to compensate for the dependence of serum CC16 concentrations on daytime. Opposite to our results, reduced CC16 concentrations around noon compared to the CC16 concentrations at 7 a.m. were described. In our study population, CC16 concentrations around noon post-ES were elevated compared to pre-ES. But blood samples pre-ES were taken earlier in the morning before the start of ES (5 a.m.), so the CC16 concentrations could be lower than at 7 a.m. As shown for these 18 subjects CC16 concentrations showed interindividual and intraindividual variations (Helleday et al. 2006), so a larger collective like ours with 672 subjects may be less confounded by such variations. Ideally, the circadian rhythm of CC16 should be further investigated in a larger cohort, including night shift workers, to monitor possible influences of different times of day.

### Atopy

Single and combined effects of exposures to allergens and DE were examined for 18 atopic subjects in an exposure chamber study by sampling bronchoalveolar lavage fluid (BALF) and plasma (Biagioni et al. 2016). The reduction of CC16 in BALF was significant after challenge with DE, but not with allergens. CC16 in plasma 4, 24, and 48 h after exposures showed no significant change, but a diurnal variation. Comparable to this, in our study atopy showed no significant effect on serum CC16 shift-differences in the model (Table 4).

### Strengths and limitations of the study

The strengths of this study are the simultaneous personal measurements of work-related exposures to NO, NO_2_, CO and EC-DPM in a high number of 672 employees over an entire work shift in combination with the medical examination before and after this ES or MS. In addition, the high participation rate should be mentioned, as 575 of them were underground workers, who represented 81% of all underground workers at this mining site. A limitation is the higher smoking consumption of the reference group in the facility above ground, where workers could smoke at least during breaks, in contrast to the workers underground, where smoking was strictly prohibited. We accounted for this issue by applying multiple linear regression modeling.

## Conclusion

In never smokers, an acute damaging effect on the small respiratory tract during a work shift in potash mining is unlikely based solely on the measured work-related exposures. However, in current smokers, there appears to be a combined inflammatory effect of active smoking and work-related exposure. Thus, in terms of effective prevention in occupational health and safety, smoking cessation is strongly recommended in smoking potash miners.

## Data Availability

The raw data generated and analyzed for this study are not publicly available as they include confidential personal medical and corporate data. As required by contract, the final dataset is, therefore, held by a trustee, the German Social Accident Insurance Institution for the Raw Materials and Chemical Industry (BG RCI) (Heidelberg, Germany).
